# History of Maturation of Prokaryotic Molybdoenzymes—A Personal View

**DOI:** 10.3390/molecules28207195

**Published:** 2023-10-20

**Authors:** Axel Magalon

**Affiliations:** Aix Marseille Université, CNRS, Laboratoire de Chimie Bactérienne (UMR7283), IMM, IM2B, 13402 Marseille, France; magalon@imm.cnrs.fr

**Keywords:** molybdenum enzyme, chaperone, biogenesis, prokaryotes, specificity, promiscuity

## Abstract

In prokaryotes, the role of Mo/W enzymes in physiology and bioenergetics is widely recognized. It is worth noting that the most diverse family of Mo/W enzymes is exclusive to prokaryotes, with the probable existence of several of them from the earliest forms of life on Earth. The structural organization of these enzymes, which often include additional redox centers, is as diverse as ever, as is their cellular localization. The most notable observation is the involvement of dedicated chaperones assisting with the assembly and acquisition of the metal centers, including Mo/W-bisPGD, one of the largest organic cofactors in nature. This review seeks to provide a new understanding and a unified model of Mo/W enzyme maturation.

## 1. Introduction

Molybdenum is the only second-row transition metal required by most living organisms to form part of the active site of enzymes, with the notable exception of many unicellular eukaryotes including parasites, most yeasts and free-living ciliates [1,2]. To date, more than 50 Mo-containing enzymes have been purified and characterized, but the metagenomic and metalloproteomic era is expected to reveal the existence of other types of enzymes with unprecedented organization [3,4]. Remarkably, the similarity between Mo and the third-row transition metal tungsten makes it an element also found in the active site of related enzymes; here, I refer to the review written by Fred Hagen in this special volume entitled “Molybdenum and Tungsten Enzymes—State of the Art in Research” on the biochemistry of tungsten [5]. In the remainder of this review, for the sake of simplicity, I will concentrate on Mo because the same principles apply to the assembly of an enzyme containing a Mo or W cofactor. This Special Issue aims to bring together a collection of historical articles that offer a personal perspective on the field of Mo/W chemistry, written by individuals who have played a significant role in its development. My article aims to provide a personal account of the development of the field—in this case, the assembly of prokaryotic molybdoenzymes—highlighting the key stages of discovery and proposing a unified model.

To gain biological activity, Mo must be bound to a special cofactor. All Mo-dependent enzymes, excluding bacterial nitrogenase, utilize a molybdenum cofactor (Moco) that consists of a mononuclear Mo atom coordinated by the *cis*-dithiolene moiety of either one or two organic molecules, pyranopterins (PPTs), at their catalytic site [6]. The different coordination patterns of the metal ion allowed the categorization of these enzymes into three large and mutually exclusive families exhibiting distinct protein folds: the sulfite oxidase (SO), the xanthine oxidase (XO), and the dimethyl sulfoxide (DMSO) reductase families. Members of all three families can be found in prokaryotes, whereas only a limited number of enzymes belonging to the XO or SO families occur in eukaryotes [2]. Importantly, the DMSO reductase family, whose members have a Mo/W atom coordinated by two guanosine-substituted PPTs, also has the greatest diversity in terms of subunit structure and/or composition (for reviews, see [7,8,9]). As early as 2013, we recommended the use of the “Mo/W-bisPGD” denomination instead of “DMSO reductase” for a safe description of the actual recognized diversity of this large enzyme family found exclusively in prokaryotes [1,8,10]. This denomination intentionally excludes the potential additional diversity, such as that associated with the absence of a molybdenum cofactor (i.e., Moco) in related protein folds. This situation has long been recognized in complex I [11,12] or alternative complex III [13,14] and has recently been surveyed by John Stolz and colleagues [4].

**Figure 1:**
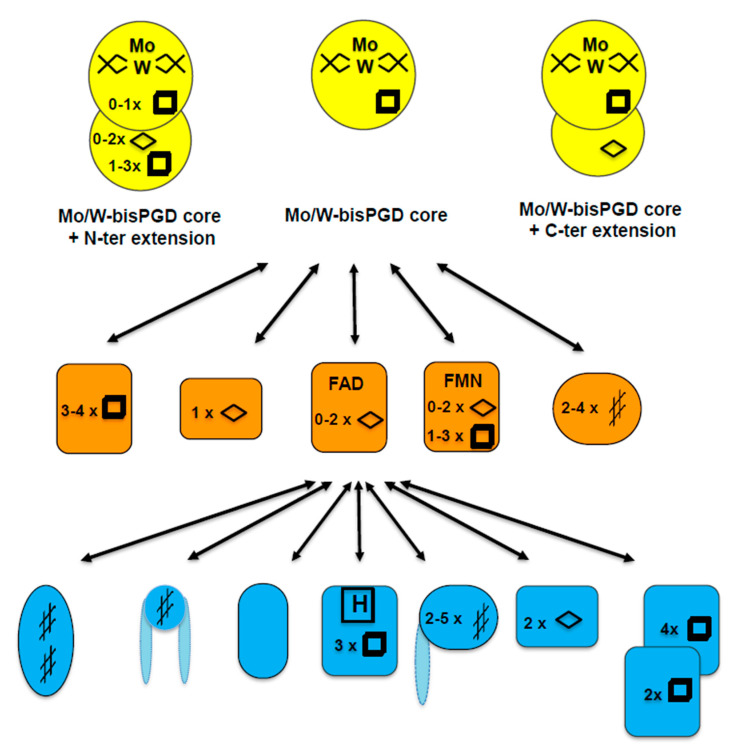
Schematic description of the modularity within the Mo/W-bisPGD enzyme superfamily. Top: catalytic subunit. The Mo/W-bisPGD-containing subunit contains a [4Fe-4S] cluster (represented by an empty square symbol), with rare exceptions, and may have N- or C-terminal extension that houses additional FeS clusters (represented by an empty square symbol for a [4Fe-4S] cluster and an empty diamond symbol for a [2Fe-2S] cluster). Middle: Electron transfer subunit. Different modules are distinguished by their sequence and structural diversities. In addition to FeS clusters, flavins or *c*-type hemes (represented by the diese symbol) can be found. Bottom: The electron entry/exit subunit. This component is the most variable element of the Mo/W-bisPGD enzyme superfamily. Again, this module can be of different types and can accommodate FeS clusters, *b* or *c*-types hemes, H-clusters or no additional cofactor at all. (Adapted from Magalon et al. [17].)

The Mo/W-bisPGD structural module serves multiple purposes in life, especially in energy harvesting, where it can convert a variety of molecules, ranging from simple inorganic molecules like CO_2_ to more complex xenobiotics molecules such as ethylbenzene, chlorate and acetylene [8]. To this end, Mo/W-bisPGD enzymes mostly catalyze redox reactions, either with oxygen or sulfur atom transfer reactions or oxidative hydroxylation (for reviews, see [15,16]). Remarkably, the Mo/W-bisPGD enzyme superfamily exhibits an extraordinarily diverse molecular organization, with the only common subunit being the catalytic subunit. Enzymes in this family can be monomeric, dimeric (with an additional subunit acting as an electron transfer module), or trimeric (with an additional subunit acting—often but not always—as a connection to the membrane quinone pool) (Figure 1).

Other modes of organization include an Mo/W-bisPGD module within a multienzyme complex, as demonstrated in recent years by the resolution of the structure of formylmethanofuran dehydrogenase [18], hydrogen-dependent CO_2_ reductase [19] and formate hydrogenlyase [20]. In addition to Moco, the catalytic subunit can contain up to five iron–sulfur clusters, whereas genomic analysis predicts the existence of more complex situations involving flavin as an additional cofactor. This diversity in the nature of cofactors increases as one moves away from the catalytic site, with the presence of iron–sulfur clusters, *b*- or *c*-type hemes, and flavins in highly variable folds. In this respect, the Mo/W-bisPGD superfamily of enzymes is one of the most prolific in the field of bioenergetics. Bioenergetics is understood to consist of a “construction kit”, with a very limited number of basic building blocks [21,22,23,24] resulting in complex modern oxidoreductases that underly the multiplicity of metabolic pathways observed in nature. These enzymes are thought to have evolved through the repeated duplication, recruitment and diversification of basic blocks. Given that all life on Earth is driven by electron transfer (i.e., redox) reactions, these enzymes are excellent subjects of study. The main challenge is to uncover the universal rules that govern the “construction kit” and that are linked to the architecture, assembly and catalytic mechanisms of proteins and complexes. It is not surprising that the study of this family of enzymes has been the Ariadne’s thread of my research for many years. In the context of this review, the Mo/W-bisPGD enzyme superfamily offers the opportunity to explore the existence of common principles or rules governing their assembly. After reviewing our current knowledge on the assembly of Mo/W-bisPGD enzymes, subdivided according to their level of complexity and specific requirements, I will attempt to describe a unified model.

In the pre-genomic era, the first indication of the existence of an additional factor essential for the activity of a Mo/W-bisPGD enzyme came from studies aimed at identifying components of the Moco biosynthetic machinery. The enzyme in question was *Escherichia coli* nitrate reductase, the tool of choice for genetic studies because of its ability to reduce chlorate to toxic chlorite. In the early 1990s, G. Giordano’s team in Marseille identified the existence of a protein factor called Factor X. This factor was distinct from the known actors in Moco biosynthesis and proved to be essential for the in vitro formation of an active nitrate reductase complex from a *chlB* strain lacking what would later be defined as the final step in Moco biosynthesis [25]. During this period, in collaboration with Marie-Andrée Mandrand-Berthelot, the group also identified *fdhD* and *fdhE* genes as essential for the activity of formate dehydrogenase complexes in *E. coli* [26,27,28]. A few years later, Tracy Palmer joined Giordano’s group as part of a short-term EMBO fellowship and the identity of the enigmatic Factor X turned out to be the NarJ protein encoded in the *narGHJI* operon containing the structural genes for the nitrate reductase complex [29]. In both systems, FdhD, FdhE and NarJ were recognized as not being part of the final enzyme assembly but as accessory proteins whose function is unclear. In 1998, while completing my PhD in Giordano’s team, I participated in a study that uncovered the true function of NarJ (IPR003765) as a dedicated chaperone that binds the catalytic subunit NarG to unequivocally facilitate the insertion of Moco [30]. The apparent contradiction with an earlier report indicating the presence of Moco in the inactive nitrate reductase issued from a *narJ* strain [31] was due to a rather qualitative fluorescence detection method for oxidized Moco derivatives. A few months later, the same team unveiled that the *torD* gene, while not being essential, encodes the chaperone for the trimethylamine *N*-oxide reductase from *E. coli* [32]. Rapidly, an analogy has been drawn with other oxidoreductases whose assembly and acquisition of metal cofactors involve accessory proteins distinct from general molecular chaperones [33,34]. As a result, I acquired expertise in this field through a post-doctoral internship at August Böck’s laboratory where I worked on the assembly of [NiFe] hydrogenases in *E. coli* prior to returning to Marseille. The NarJ protein has been identified as a prototype of accessory proteins in prokaryotic Mo-enzymes due to its essential nature. Following this discovery, many research groups have reported the involvement of similar proteins in the synthesis of active Mo-enzymes, including dimethyl sulfoxide reductase [35,36,37], periplasmic nitrate reductase [38,39,40], putative tetrathionate reductase [41], steroid C-25 hydroxylase [42] and xanthine dehydrogenase [43]. With the advent of the genomic era, numerous operons encoding for prokaryotic Mo-enzymes have been identified and include a gene encoding for a putative dedicated chaperone (for review, see [9,44,45]). A consensus has now emerged regarding the general role of chaperones dedicated to Mo/W-bisPGD enzymes in assisting Moco insertion.

As reviewed by Magalon et al. [17], the modular organization of many Mo enzymes involves multiple subunits, with the electron entry/exit subunit being the most variable (Figure 1). With or without redox-active cofactors, integrated or not into the membrane, this module is of fundamental importance for the integration of the enzyme into the bioenergetic chain in which it is involved. Enzymes can form cytoplasmic or periplasmic subcomplexes in the absence of membrane-anchoring subunits. These subcomplexes retain oxidoreductase activity but are not any longer linked to the electron transfer chain. This implies that attaching enzymes to the membrane via their anchor subunits is usually the last step in assembly. In prokaryotic cells, Mo-enzyme synthesis and assembly is complex, requiring synthesis of subunits, assembly, incorporation of metal/organic cofactors in the cytoplasm and membrane anchoring. In the case of periplasmic Mo-enzymes, the assembly and metal cofactor incorporation steps occur in the cytoplasm prior to the translocation of the folded substrate across the inner membrane via the Tat apparatus [46,47,48]. Importantly, it soon became clear that the coexistence of an iron–sulfur cluster and Moco in the catalytic subunit of members of the Mo/W-bisPGD enzyme superfamily likely arose early in evolution, paving the way for a recent evolution involving the loss of the FeS cluster [4,12]. Based on this observation, the NarJ chaperone was thought for a long time to be the archetypal chaperone for the assembly of a wide variety of complex multimeric enzymes before it was recognized as representing a wider family of chaperones [49,50]. Finally, most of the available information on the assembly of prokaryotic Mo-enzymes concerns members of the Mo/W-bisPGD and XO families, as described below. Indeed, no enzyme-specific chaperones have been found for aldehyde oxidoreductase and sulfite oxidase family enzymes, although the presence of an FeS cluster within the catalytic subunit in some cases [51,52,53] or the periplasmic location of the enzyme [54] suggests the involvement of enzyme-specific chaperones.

## 2. Maturation of Mo/W-bisPGD Enzymes Illustrated by the Cytoplasmic and Multimeric Nitrate Reductase

Several members of the Mo/W-bisPGD superfamily were likely present in the last common universal ancestor (LUCA), with the common presence of an FeS cluster and the Moco in the catalytic subunit, as suggested from both phylogenetics and geochemistry data [4,12,55]. A general characteristic of this enzyme family is a multisubunit organization aimed at connecting the catalytic module to redox partners. As such, cytoplasmically oriented membrane-bound NarGHI-type nitrate reductase (i.e., nNar) has long been considered as a general model for the assembly of several Mo/W-bisPGD enzymes. This enzyme is widespread in bacteria and archaea and is proposed to have existed in LUCA [55]. The genomic organization typically includes the *narJ* gene within an operon, which encodes the three other structural components. The catalytic dimer is composed of a complex between the NarG subunit, which holds the Moco and a His-coordinated [4Fe-4S] cluster (FS0), and the electron transfer subunit NarH, which coordinates three [4Fe-4S] and one [3Fe-4S] clusters [56,57]. The dimer is anchored to the membrane via the NarI subunit, which coordinates two *b*-type hemes [58], as confirmed by the resolution of the crystal structure [59].

In the absence of NarJ, a global defect in metal incorporation into NarGHI is associated with the synthesis of an inactive but stable complex mostly attached to the membrane [30,60]. EPR spectroscopy conducted in collaboration with Guigliarelli’s group in Marseille appeared to be manifold in delineating the metal cofactor content in several purified stable assembly intermediates isolated due to the inactivation of NarJ or Moco biosynthesis or the deletion of one of the NarJ-binding sites on NarG [61]. Not only are the Moco and the FeS cluster of the catalytic subunit NarG absent but also the heme b_P_ is positioned at the interface between NarGH and NarI. In total, three different types of metal cofactors were not incorporated when the chaperone was absent. To solve this apparent paradox and the unexpected stability of the resulting enzyme, one has to examine the apoNarGHI crystal structures lacking either the Mo-bisPGD (PDB ID code 1siw) [62] or both FS0 and Mo-bisPGD (PDB ID code 3ir6) [63]. In both cases, the insertion of GDP moieties at the exact positions occupied by Mo-bisPGD confers structural stability. This situation is also encountered in the case of CO dehydrogenase from *Hydrogenophaga pseudoflava* expressed from tungstate-grown cells [64] or *Rhodobacter sphaeroides* DorA protein heterologously expressed in the Mo-bisPGD deficient *mob E. coli* strain [65], both being fully loaded with corresponding nucleotides in the absence of the cofactor. How can we explain the importance of the NarJ chaperone in the acquisition of distinct types of metal cofactors? The answer lies in the definition of the binding sites on the NarG catalytic subunit.

Two distinct NarJ binding sites were mapped on the NarG catalytic subunit, one corresponding to the N-terminus [60,66]. NarJ binding to this region, which is summarized in the first 15 amino acids, is part of a chaperone-mediated quality control process that prevents the membrane-anchoring of the soluble and cytoplasmic NarGH complex before all maturation events have been completed. This is illustrated by the accumulation of a soluble apoNarGH complex in the *mob* strain (~40%), which was further enhanced upon NarJ overproduction (~80%). Prior to Moco insertion, NarJ maintains the apoenzyme in a soluble state. However, once attached to the membrane, the apoNarGH complex can no longer incorporate Moco [60]. In other words, once bound to the NarI subunit, the apoNarGH complex acquires a definitive conformation that is no longer compatible with the NarJ-assisted Moco insertion process. Such a conformation could be acquired through the incorporation of GDP molecules, as disclosed by the crystal structure of the corresponding form of the enzyme produced in a *mob* strain, which shows virtually no change in the overall fold [62]. The positioning of the N-terminus of NarG, which is embedded within the oligomeric structure of NarGHI and interacts with NarI, sheds light on the interference with membrane anchoring that takes place upon interaction with NarJ. Deletion of this region not only impacts membrane anchoring, as anticipated but still allows NarJ-assisted Moco insertion, providing additional proof for the existence of a second NarJ binding site on the core domain of NarG [60]. A thorough examination of the metal cofactor spectral signature by EPR distinguished NarJ as a crucial factor in the complete maturation of the *b*-type cytochrome NarI, guaranteeing the proper timing for the NarGH complex’s membrane anchoring [61]. The absence of the proximal heme b_P_ in the *narJ* strain can be attributed to the lack of synchronization between the maturation of NarI and NarGH components, as both hemes are inserted in the absence of the catalytic dimer and NarJ. Furthermore, the insertion of the two hemes is independent of the Moco insertion, as is the case in a *mob* strain [61,62]. This process strongly resembles the “Tat proofreading” of periplasmic metalloproteins, of which the best-studied examples are trimethylamine *N*-oxide reductase and [NiFe] hydrogenase 2 from *E. coli* [67]. The correlation between the Tat signal peptides and the N-terminus of the non-exported nNarG [49,68] is further reinforced by similarities in archaea and some bacteria, where the NarG sequence possesses a typical Tat signal peptide that is instrumental in the periplasmic localization of the NarGH complex (for review, see [8]). However, it is important to note that the analogy only goes so far, as the membrane anchoring and activity of the *Ec*NarGHI complex are not affected in a *tat* strain (Pommier and Magalon, unpublished results).

Collaborating with Berenguer’s group in Madrid led us to study the assembly of the homologous nitrate reductase complex in *Thermus thermophilus*. This complex includes an additional structural partner, the NarC protein, a membrane-anchored, periplasmic *c*-type cytochrome [69]. Our surprise was that inactivation of the *narI* and *narC* genes, which encode the two membrane-anchoring subunits, resulted in an unstable but inactive NarGH complex [70]. An identical behavior was observed in the absence of NarJ. In this thermophilic organism, the NarI and NarC proteins are as important as NarJ in assembling the complex. Given the results obtained in *E. coli*, one possible explanation for these differences could be due not only to a different contact interface with the membrane partners but also to the absence of the N-terminal end of NarG in *T. thermophilus*, recognized as a binding site for NarJ, and also of the immediate β-hairpin, which forms a twisted β-sheet with NarI.

A second NarJ binding site within the NarG catalytic subunit is responsible for the sequential insertion of the FeS cluster (FS0) followed by Mo-bisPGD, as demonstrated both in vivo and in vitro [61]. Indeed, while the lack of Moco does not preclude FS0 insertion, the absence of NarJ or substitution of cysteine ligands of the FS0 cluster prevents the insertion both of FS0 and of Moco in NarG [61,63]. In our search for Moco-driven conformational changes, we were successful in isolating and characterizing a stable prefolded state that remains competent for cofactor insertion, like that of the cytoplasmic and soluble apoNarGH complex produced in the absence of NarJ [71]. It is worth recalling that this assembly intermediate contains all four FeS clusters in the NarH subunit. Through comparative SAXS analysis of the active holoNarGH complex and the inactive one, we found that conformational changes associated with cofactor insertion are limited to one structural motif in the catalytic subunit. This motif exhibits strict conservation within members of the Mo/W-bisPGD family, with a solvent-exposed salt bridge (R108-E794 according to *E. coli* Nar numbering) being instrumental for enzyme folding upon cofactors insertion. Disruption of the salt bridge and the concomitant movement of the associated loops reproduced the conformation adopted by the apoNarGH complex produced in the absence of NarJ, while this model rise a poorer fit to the SAXS data of holoNarGH. The R108A variant produced a poorly active but stable complex with reduced metal cofactor content in the NarG subunit, reflecting a reduced interaction with NarJ. Our current understanding is that NarJ-dependent sequential cofactor insertion occurs prior to salt bridge formation, i.e., in an open conformational state of the NarGH complex. In addition, this study provides a first and crucial clue regarding the location of the second NarJ binding site within NarG, Arg108, which is involved in the interaction with NarJ [71]. Comparative analysis on the structurally and phylogenetically related NuoG subunit of complex I, exemplifying the case of Moco-less protein fold and named MopB by Wells et al. [4], confirms our working model. The strict conservation of the residues involved in the surface-exposed salt bridge in all Mo/W-bisPGD family members suggests that the same conformational changes observed in the nitrate reductase complex also occur in these other members. Despite this, the exact function of NarJ in the Moco insertion process remains unclear, despite its crucial role in authorizing the interaction of the apoenzyme with a complex of cofactor biosynthetic proteins responsible for Moco delivery [72,73].

**Figure 2:**
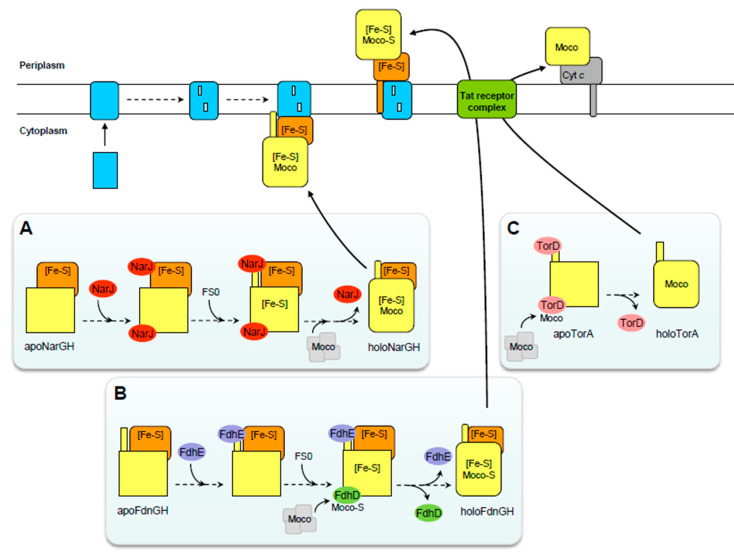
Biogenesis models of the *E. coli* nitrate reductase A, formate dehydrogenase N and the TMAO reductase complexes. These enzymes were selected to illustrate typical examples of complex multimeric enzymes (NarGHI and FdnGHI) or monomeric enzymes belonging to the Tor/Dor/Bis clade (TorAC). (**A**) panel: After the nascent chains exit the ribosome, the FeS clusters are co-translationally inserted into the NarH electron transfer subunit, forming a stable complex with NarG. This complex is kept soluble by the dynamic binding of NarJ to the remnant Tat signal peptide at the N-terminus of the NarG catalytic subunit. The binding of NarJ to the central domain of NarG maintains the protein in a state that is capable of acquiring the fifth FeS cluster. The associated conformational change allows Moco to be subsequently delivered via a Moco biosynthesis platform and with the assistance of NarJ. At the end of the cofactor insertion step, a solvent-exposed salt bridge forms, leading to the final conformation of the catalytic dimer and the dissociation of the chaperone at both sites. Please note that the stoichiometry of the chaperone–enzyme complex is still unknown. The catalytic dimer can now interact with its membrane partner subunit, a *b*-type diheme cytochrome, NarI. For most periplasmic and multimeric enzymes, as illustrated with DmsABC, the scheme is identical except that the catalytic dimer is translocated prior to interaction with its partner. (**B**) panel: An immature FdnGH complex is first formed and recognized by two distinct chaperones. It is likely that FdhE interacts with the Tat signal peptide of FdnG onto the apoFdnGH complex, preventing it from interacting with the Tat apparatus. It is expected that the insertion of the FeS cluster within FdnG occurs before the Moco insertion, whether or not this involves the participation of a chaperone. Once loaded with Moco, FdhD interacts with IscS to allow sulfuration of the cofactor and its subsequent transfer to apoFdnGH but also apoFdoGH or FdhF targets. As in the case of Nar, the chaperones dissociate from both sites upon completion of the cofactor insertion step. The mature complex can now be addressed to the Tat receptor complex for translocation across the inner membrane. (**C**) panel: The binding of the TorD chaperone to the core domain of the TorA catalytic subunit, at a similar site to the one involved in multimeric enzymes, stabilizes the folding state competent for Moco acquisition, while dynamic binding to the Tat signal peptide prevents premature interaction with the Tat receptor complex of the immature substrate. The Moco insertion step involves transient binding of the cofactor to the TorD chaperone prior to its transfer to the catalytic subunit. It is understood that Moco can also be transferred directly without the aid of a dedicated chaperone, but likely with that of molecular chaperones. The conformational change of TorA associated with cofactor insertion likely results in the release of TorD and the interaction of the Tat signal peptide with the receptor complex for subsequent translocation.

These data collectively demonstrate that NarJ plays a crucial role in the maturation of the NarGHI complex by orchestrating the steps of metal cofactor insertion, subunit assembly and membrane anchoring (Figure 2).

NarJ proofreads the insertion of metal centers into the catalytic subunit by binding to a remnant Tat signal peptide and in a coordinated manner to bind to a distant binding site on NarG. The mechanism described is similar to that of Tat substrate translocation. Additionally, the comparison with other multimeric Mo enzymes suggests that NarJ-like proteins may have similar functions, as shown in the following section and further discussed later.

## 3. Periplasmic and Multimeric Mo-bisPGD Enzymes

A number of periplasmic and multimeric enzymes of the Mo/W-bisPGD family have been genetically or biochemically characterized in different bacteria or archaea such as the selenate [74], nitrate [75], chlorate [76,77], perchlorate [78,79], arsenate (i.e., Arr) [80,81] or tetrathionate [82] reductases and the ethylbenzene [83], steroid C25 [42,84], dimethylsulfide [85] dehydrogenases or alternative arsenate oxidase (i.e., Arx) [86,87]. Given their similarity to the NarGHI or DmsABC complexes (see below) and the presence of an additional gene encoding a NarJ-like chaperone protein in the corresponding operons [50,55], it is reasonable to speculate that the folding and assembly of these Mo-enzymes will follow a similar pattern (Figure 2). The periplasmic and multimeric arsenite oxidase (Aio) and polysulfide (Psr) enzymes are exceptions as they do not have an additional gene encoding for a chaperone in their respective operons [88,89,90]. However, the presence of a Tat signal peptide and an FeS cluster together in the catalytic subunit suggests that these enzymes may require assistance from an unidentified chaperone, which may be located elsewhere in the genome. Two heterotrimeric complexes in *E. coli*, the DMSO reductase DmsABC and the formate dehydrogenase FdnGHI share similarities with the NarGHI complex in terms of subunit and redox cofactor composition. Extensive analysis of their assembly by several groups revealed both common principles and distinct maturation requirements, as detailed below.

### 3.1. The Case of the DMSO Reductase: A Similar Situation to Nar

In 2001, Oresnik et al. reported that DmsD is crucial for the formation of a fully functional *E. coli* DmsABC complex [36]. The *dmsD* gene, previously known as *ynfI*, is part of the *ynf* operon, which encodes putative Tat-targeted selenate reductases in both *E. coli* and *Salmonella enterica* [47,91]. It is noteworthy that DmsD is responsible for both the folding and assembly of the DmsABC complex and the two putative selenate reductases in *E. coli* [91,92], contrasting with the usual exquisite specificity of these chaperones. As discussed later in this review, several groups have reported that the chaperone has the ability to recognize multiple Tat signal sequences or partners, raising the question about how it recognizes them. Here, the strong sequence similarity of the corresponding catalytic subunits (DmsD, YnfE, YnfF), which coordinate both a FeS cluster and a Mo-bisPGD, may explain such behavior.

DmsD was identified as the first protein to bind a Tat signal peptide, namely, the one from *E. coli* DmsA with a *K*_D_ of ~220 nM [36,93]. This groundbreaking discovery led to a better understanding of the control necessary for the export of folded and metal-loaded Tat substrates. The removal of the DmsA signal peptide leads to the production of a less stable yet soluble and cytoplasmically active DmsAB complex [94]. It is likely that the DmsA variant benefited from the action of DmsD on a second binding site for metal cofactor acquisition. Thus, it is necessary for DmsD to act on both the signal peptide and a second site of the DmsA protein, similar to NarJ, in order to achieve productive synthesis of DmsABC. The same principle also applies to the requirement for FeS insertion into the DmsA catalytic subunit prior to Moco insertion [95]. Despite the absence of a thorough biophysical characterization of the complex formed in the absence of DmsD, several indirect lines of evidence suggested that the Moco-free enzyme complex was stable and appropriately localized [96,97]. Furthermore, translocation of the DmsAB catalytic dimer occurs in the absence of the membrane anchor subunit DmsC [98]. X-ray structural analyses of DmsD from *E. coli* (PDB ID codes 3efp and 3cw0) [99,100] and *S. typhimurium* (PDB 1s9u) [101] indicate that it has an all α-helical architecture, similar to NarJ and TorD. Structural plasticity is exhibited by DmsD, with the presence of multiple fold forms that would interconvert with pH [93,102] or a disordered loop in the X-ray data adjacent to an elongated hydrophobic groove [101], which may enhance its ability to interact with multiple binding sites on the partner. This has been demonstrated for NarJ [60,61]. In the context of the translocation of folded substrates, TatBC proteins act as a receptor complex that recognizes the signal peptide of the substrate protein [103], while Turner’s group further hypothesized that the chaperone could interact with the Tat receptor complex during substrate addressing [104]. This hypothesis was based on the observation that DmsD interacts with TatB and TatC located in the inner membrane [105,106,107]. However, this issue remains controversial as the TorD-related chaperone does not bind Tat structural proteins [108]. DmsD has also been shown to interact with general chaperones and several Moco biosynthesis proteins, expanding its interactome and raising questions about the universality of this property and its feasibility [109].

One may think that the assembly of multimeric Mo/W-bisPGD enzymes is well understood by these examples, NarJ and DmsD being equivalent prototypes of dedicated chaperones. As developed below, peculiar situations are encountered in the periplasmic nitrate reductases (Nap) and formate dehydrogenases (i.e., FDHs) with the participation of chaperones that do not belong to the NarJ family (IPR003765). Interestingly, a phylogenetic tree deduced from comparative sequence analysis of the catalytic subunit of members of the Mo/W-bisPGD family reveals that Nap and FDH form two separate but closely related phyla distant from the others [55,110]. They also share the presence of an additional sulfur ligand in the axial position of the Mo/W atom (for reviews, see [111,112]). This subtle distinctive characteristic appears to be key with the emergence of different requirements for assembly.

### 3.2. The Case of Formate Dehydrogenases: Two Distinct Chaperones and a Sulfuration Step of the Cofactor

*Escherichia coli* produces three types of FDHs, two of which are periplasmic membrane-bound respiratory complexes, the nitrate-inducible FdnGHI and the cryptic FdoGHI [113]. These contain typical Tat signal peptides on their catalytic subunits. The third type of FDH, composed of a single cytoplasmic subunit (FdhF), forms part of the formate hydrogenlyase complex [114]. All three contain a [4Fe-4S] cluster in addition to the Mo-bisPGD cofactor in their catalytic subunit. Genetic studies have shown that both *fdhD* and *fdhE* genes, located adjacent to the *fdo* operon, are critical for the formation of active FDHs. Specifically, *fdhE* is restricted to periplasmic ones [26,27,28,115,116]. Furthermore, bioinformatic analysis of the genomic organization of *fdh* operons indicates the nearly systematic proximity of an *fdhD* gene, with some exceptions in archaea, while *fdhE* is only associated with operons encoding putative periplasmic FDHs.

The two chaperones, FdhD (IPR003786) and FdhE (IPR024064), stand out with their lack of structural similarities to other chaperones. FdhD forms a dimer and has a mostly helical architecture, as observed in the crystal structure of FdhD from *E. coli* [117] and *Desulfotalea psychrophila* (PDB ID codes 4PDE and 2PW9, respectively). The structure of *E. coli* FdhD in complexes with GDP reveals the presence of a structural motif known to interact with nucleotides: a α/β-Rossmann fold. Both crystal structures show dimers connected by a small tunnel between two opposite faces, with one face able to accommodate two molecules of GDP through a set of conserved residues. The opposing face features two disordered loops containing catalytic cysteine residues. FdhE is an iron-binding rubredoxin that contains four conserved CX2C motifs, which are crucial for its stability and biological function [118]. The crystal structure of FdhE from *Pseudomonas aeruginosa* PAO1 (PDB ID code 2fiy) reveals that each pair of CX2C motifs located in disordered loops coordinate an iron atom, whose role is yet to be determined. Since FdhE is only necessary for the activity of formate dehydrogenases located in the periplasm, it can be inferred that it plays a role in the transport event, while FdhD is responsible for inserting metal centers. However, if FdhE has been shown to interact both with the FdnG and FdoG catalytic subunits, a direct role in Tat proofreading has not yet been demonstrated [118]. Intrigued by the existence of a distinctive class of dedicated chaperones for FDHs, we decided to focus on FdhD. A major breakthrough came with our study demonstrating that FdhD functions as a sulfurtransferase between the major cysteine desulfurase IscS and FdhF in *E. coli* [119]. Notably, the activity of FdhF was shown for the first time to be sulfur-dependent. In the absence of FdhD, the inactive but stable FdhF protein contains both metal centers, excluding any absolute role in cofactor insertion. We reported the in vivo detection of Mo-bisPGD binding on FdhD and proposed a working model in which FdhD simultaneously ensures the sulfuration of Moco and its subsequent protected transfer to apo-formate dehydrogenases [117,119]. Owing to the nucleotidic character of the Mo-bisPGD cofactor and the high affinity of FdhD for GDP (*K*_D_ ~364 nM), we proposed that FdhD would bind it through the nucleotide-binding motifs disclosed by the structure. Therefore, the working model predicts that FdhD binds the Mo-bisPGD cofactor and ensures its sulfuration via a sophisticated mechanism by which inorganic sulfur is transported from IscS on one side of the dimer to Moco on the other side via the tunnel (Figure 2). Two cysteine residues in the disordered loop are involved in sulfur transfer with Cys-121 (*E. coli* numbering), which is essential for sulfur transfer from IscS to Moco as well as in FDH activity. The sulfurated cofactor is then transferred to the target enzymes. This model explains the presence of inorganic sulfur at the metal ion coordination sphere of Mo or W-formate dehydrogenases and its essential role in enzyme reactivity [120,121]. An interesting case is FdhD (also named FdsC) in *Rhodobacter capsulatus*, which shares common features with *E. coli*’s counterpart, except that the two cysteine residues present in the loop are not essential for FDH activity [122,123]. Sequence alignment analysis revealed that, in some cases, FdhD contains one or no cysteine residues in the loop [117,124]. A prominent example is that of the euryarchaeota *Methanococcus maripaludis*, in which FdhD has conserved the ability to bind Moco as judged by the conservation of the nucleotide-binding motif, but does not possess cysteine residues in the loop. Importantly, this archaeon appears to lack a cysteine desulfurase and an alternative source of sulfur to cysteine is proposed for FeS biogenesis [125,126]. The SUF-like minimal system, SMS, is proposed to ensure FeS biogenesis in the absence of a cysteine desulfurase in this archaeon [127]. Based on the observation that Moco insertion proceeds even in the absence of the cysteine residues of FdhD, it is tempting to speculate that sulfuration proceeds via a different mechanism, while FdhD ensures Mo-bisPGD insertion into the target enzymes. Notably, in many cases where bacterial FdhDs have no cysteine residues in the loop, the organisms harbor the minimal iron-sulfur machinery, MIS [127]. Another intriguing case is the biogenesis of a formate dehydrogenase in *Campylobacter jejuni*, which requires the participation of FdhM, a TorD homologue, encoded in the operon containing the structural genes but also *fdhD* [128]. What remains unclear is the respective roles of FdhM and FdhD in the assembly process of the periplasmic FdhABC. Notably, the absence of FdhM has no impact on Tor activity in *C. jejuni*. One may infer that FdhM acts as a proofreader for the FdhAB complex, binding to the Tat signal peptide of FdhA prior translocation, while FdhD is responsible for inserting sulfurated Moco.

### 3.3. The Case of Periplasmic Nitrate Reductases: A Distinct Chaperone, a Sulfuration Step of the Cofactor and Open Questions

The folding and assembly of periplasmic nitrate reductases involves two cytoplasmic proteins, NapD and NapF, which do not have any sequence or structure similarity with other dedicated chaperones. In the absence of NapD, the enzyme is inactive and fully degraded [39,129]. Sargent’s group reported that *Ec*NapD has a ferredoxin-type fold and is involved in Tat signal peptide binding [129]. NMR studies have demonstrated that the Tat signal peptide of NapA adopts a helical conformation during complex formation with NapD and primarily binds through hydrophobic contacts [130]. These observations were later confirmed using site-directed spin labeling coupled with EPR spectroscopy by the same group [131]. Although the structures of NarJ and NapD are unrelated, a similar situation has been observed with the remnant Tat signal peptide of *Ec*NarG, which also adopts a helical conformation [132]. Analysis of the binding process by ITC revealed the existence of two distinct populations of NapD at pH 7.5, with a minor population (35%) having an apparent *K*_D_ of ~3 nM and a major one (64%) having a much higher *K*_D_ of ~140 nM [130]. No molecular explanation for such a phenomenon in NapD is provided, but the protonation of a specific residue within the binding pocket of NarJ was shown to be responsible for the variation of binding constants (Zakian and Magalon, unpublished results). Altogether, these similarities suggest a common mode of action. If the interaction between NapD and the NapA signal sequence is responsible for the proofreading activity of the metalloprotein before translocation, what about the process of metal center acquisition by NapA? Who ensures the sulfuration of the Mo-bisPGD? However, there are few answers to these questions. Dow et al. [131] reported that, in the absence of the Tat signal sequence, a low-affinity complex exists between NapA and NapD, leading to the hypothesis that NapD has the capacity to bind to the core domain of NapA. Turner’s in vitro studies did not find evidence of NapD binding to the mature region of NapA [133], which left the question unresolved.

On the contrary, NapF is not always present in *nap* operons such as *Campylobacter* species or *Paracoccus denitrificans* questioning its actual role. While impacting marginally the activity in *E. coli* [134] or in *Wolinella succinogenes* [135], the absence of NapF in *R. sphaeroides* results in a dramatic decrease of activity correlated with the destabilization of NapA [136,137]. Interestingly, NapF harbors four conserved tetra-cysteine motifs allowing for the coordination of four labile [4Fe-4S] clusters. In vitro reconstitution of the FeS cluster in *Rs*NapA was shown to require NapF and interpreted as a direct role of this cytosolic protein in NapA maturation prior to export via the Tat translocon [137]. Direct evidence for complex formation between NapF and NapA in *E. coli* provided additional support to this functional role [138]. As suggested by several authors, one may envision that the NapF function can be duplicated to some extent, providing an explanation not only for its loss during evolution but also for conflicting results among the studied organisms. The same line of thought can be drawn for the non-conserved *napL* gene whose deletion has only a moderate impact on Nap activity in *C. jejuni* or *W. succinogenes* [40,139].

In any case, the identity of the protein in charge of the sulfuration of the Moco in Nap remains unknown. While in the first instance, one may consider that the mechanism is enzyme-specific, as described for FdhD or for the *R. capsulatus* xanthine dehydrogenase that requires XdhC for sulfuration [140,141] (see below), none of the identified accessory proteins seem to endorse such a role. Alternatively, the sulfuration mechanism for Nap could rely on a promiscuous system as in eukaryotes for the sulfuration of xanthine dehydrogenase and aldehyde oxidase (for review in this Special Issue, see [142]).

In this section, we observed that the assembly and correct localization of multimeric Mo-bisPGD enzymes require specific requirements to be satisfied through the participation of a dedicated chaperone that interacts with the N-terminus of the catalytic subunit (i.e., the Tat signal peptide remnant or not) to proofread the complete metal center acquisition within the catalytic subunit, which involves sequentially acquiring an FeS cluster followed by the cofactor. This process requires the binding of the chaperone to a distinct site, which has only been identified in the case of NarG. As a result, NarJ and DmsD share common properties due to the nearly identical assembly requirements of their corresponding enzymes. While the participation of a related dedicated chaperone (often encoded in the operon containing the structural genes) has been proposed for a number of other cytoplasmic or periplasmic multimeric Mo-bisPGD enzymes listed above, in the absence of experimental investigation of their maturation process, we can only infer that their corresponding chaperones will follow the same trend (Figure 2). Interestingly, two separate classes of Mo-bisPGD have been shown to involve distinct chaperones while sharing the need for a sulfuration step of their Moco. This has been best illustrated with *E. coli* and *R. capsulatus* formate dehydrogenases, where FdhD has been demonstrated to coordinate sulfuration of the cofactor and its protected transfer to the target enzymes, which can be multiple, as in *E. coli*. In contrast, cofactor insertion was not impaired in the absence of FdhD, which contrasts with NarJ. However, a caveat is that the *fdhD* gene is absent in some organisms that express formate dehydrogenases. Another open question is how the likely sequential insertion of FeS and sulfurated Moco into NapA proceeds in the case of periplasmic nitrate reductases, where the participation of a proofreading chaperone, NapD, has been observed to bind to the Tat signal peptide.

## 4. Periplasmic and Monomeric Mo-bisPGD Enzymes

The biogenesis of multimeric Mo-enzymes harboring a FeS cluster and a Mo-bisPGD in the catalytic subunit appears to be intricate and necessitates a dedicated chaperone. What about monomeric Mo-enzymes (TorA/DorA/BisC) that have Mo-bisPGD as the sole prosthetic group and are exclusively present in some phyla [12,22]? Among these, BisC is the only non-exported Mo-enzyme and it is unclear whether Moco insertion is assisted by a chaperone. In contrast, the production of an active and periplasmic TorA/DorA enzyme depends on the action of a chaperone as detailed below.

In *E. coli*, the reduction of trimethylamine *N*-oxide (TMAO) is primarily ensured by a periplasmic respiratory system encoded by the inducible *torCAD* operon [143,144]. The last gene of the *tor* operon, *torD*, encodes a cytoplasmic protein with sequence similarities to NarJ and DmsD (IPR023069) [49]. Pommier et al. reported that the absence of TorD results in twofold less but still active and correctly periplasmically localized TorA in *E. coli* [32]. Additionally, the produced enzyme in a *torD* strain is subject to proteolysis under thermal stress conditions, Moco deficiency or molybdenum starvation [145,146,147]. The relative stability of apoTorA in *E. coli* in these specific conditions results from a protective action of *Ec*TorD counteracting the degradation of the enzyme by impairing the binding of the Lon protease [148]. In contrast, instability and loss of activity are observed with *R. capsulatus* DorA or *Shewanella oneidensis* TorA, in the absence of their respective DorD and TorD chaperones [35,149]. These observations differ from the multimeric nitrate reductase, where the absence of NarJ does not significantly affect the stability and the cellular localization of the enzyme complex [30,60]. The initial formation of a stable apoNarGH complex partially loaded with metal centers in NarH prior to the action of NarJ as compared to the monomeric Moco-only DorA/TorA enzymes is likely an explanation. In 2000, the situation became even more complex, with two parallel studies questioning the need for a chaperone during Mo-bisPGD insertion in Dor/Tor enzymes or the participation of a promiscuous chaperone that had yet to be identified. The first study, conducted by Temple et al., found that apoDorA from *R. sphaeroides* could acquire up to 73% of Moco using an in vitro reconstitution assay, without the aid of a chaperone [65]. The stability of the *Rs*DorA protein purified in the absence of Moco likely resulted from a full complement of nucleotides (GMP or GDP) working as structural surrogates of the cofactor. The second study, conducted by Gon et al., revealed that the cryptic periplasmic TorZY enzyme complex from *E. coli* was active without TorD [150]. A definitive answer to the function of the TorD protein was provided by Iobbi-Nivol’s group in *E. coli* [151]. TorD was confirmed to interact with apoTorA and enhance Moco insertion, using an in vitro reconstitution assay.

TorD interacts with two distinct sites of the TorA enzyme, one of which is the Tat signal peptide located at the N-terminus [67,152]. This property is a general principle for this class of chaperones (IPR036411). Genest et al. reported that TorD protects the TorA signal peptide regardless of the presence of Moco and or the Tat translocase [146]. It is currently unclear whether TorD binds to the signal peptide to monitor the folding and assembly of the substrate or if it hinders the export kinetics, allowing the Moco insertion process to be complete. Using a translational fusion between the TorA signal peptide and GFP, contrasted results were obtained. Li et al. [153] reported that TorD stimulates the translocation of the fusion, in support of the reported protective role of the chaperone, while Bageshwar et al. [108] showed that TorD inhibited the binding of the fusion to the membrane but only moderately impacted transport efficiency in agreement with the dynamic nature of this interaction. In parallel, a structural investigation of the TorD protein revealed that the one from *Shewanella massilia* forms multiple and stable oligomeric species and that both the monomeric and dimeric species bind TorA [154]. Only the dimeric form has been crystallized, revealing domain swapping between the two monomers having an all α-helical fold (PDB ID code 1n1c) [155]. Nevertheless, the biological significance of these oligomers reported for several TorD homologs [156] is still under debate as no gain of function in terms of binding affinity, usually encountered through domain swapping, has been observed on dimerization. For instance, only the TorD dimer exhibits a low GTPase activity and a weak affinity for GTP (*K*_D_ ~ 370 µM), despite the absence of classical nucleotide-binding motifs [152,157]. Moreover, most residues involved in GTP binding are not conserved [50]. If the role of nucleotide binding or hydrolysis in modulating the interaction between the signal peptide and TorD remains unclear, the monomeric form is sufficient to bind both TorA sites [152,158]. Bageshwar et al. [108] not only established that *Ec*TorD is predominantly a monomer under physiological conditions but also that its main function via signal peptide binding is to protect it from degradation during folding and Moco acquisition.

A second TorD-binding site was established on the core of the TorA enzyme, leading to a conformational change of the latter [32]. Using an in vitro system, Mo-bisPGD insertion within apoTorA was shown to be facilitated by the presence of TorD, even in the absence of the signal peptide [156]. At the same time, Jack et al. confirmed the existence of two TorD-binding sites on TorA through the use of signal peptide-swapped fusions [67]. To reveal the second binding site, SAXS experiments were conducted on a complex made between *Ec*TorD and apoTorA with or without its Tat signal peptide [158]. While these studies did not provide sufficient information about the location of the binding area, they confirmed that a stable complex between the chaperone and its target can be made independently of the signal peptide. Several studies also reported TorD variants impaired in either TorA signal peptide binding or Moco insertion, indicating that two distinct regions of TorD are involved in recognizing the two TorA-binding sites [67,159]. Another level of complexity was introduced with the demonstration that *Ec*TorD interacts with Moco biosynthesis components, including MobA and Mo-PPT [159]. The authors proposed that TorD serves as a platform between the final stage of Mo-bisPGD synthesis and its integration into TorA, although it is not essential. This raises the question of whether other chaperones of Mo/W-bisPGD enzymes have a similar function, which warrants further investigation, particularly with regard to Moco-only enzymes, which do not necessarily require a dedicated chaperone for folding.

## 5. Maturation of Other Prokaryotic Mo/W-Enzymes

The two other families of Mo/W-enzymes found in prokaryotes are xanthine oxidase and sulfite oxidase. The difference between them is that xanthine oxidase contains a sulfur atom on the Mo coordination sphere, while sulfite oxidase does not. A well-established post-translational mechanism for Moco sulfuration has been studied in detail that involves a Moco-binding protein interacting directly with a cysteine desulfurase to ensure the sulfuration step of the cofactor and its protected transfer to the apoenzymes. This mechanism is conserved from bacteria to eukaryotes. In eukaryotes, the two components are combined into a single polypeptide called Moco sulfurase [160], while in prokaryotes, a dedicated chaperone operates together with a cysteine desulfurase [140]. The best-characterized dedicated chaperone for members of the xanthine oxidase family in prokaryotes is the *Rhodobacter capsulatus* XdhC protein, which is essential for the folding and assembly of *R. capsulatus* XDH (for review, see [161]). A description of its mode of action will be developed below. However, as mentioned previously, no chaperones have been identified for members of the SO family, leaving open how metal acquisition, folding and even translocation steps of the metal-loaded complex are achieved as best illustrated with the MsrPQ complex in *E. coli* [54,162].

Leimkühler’s group has been actively studying the functional role of XdhC or XdhC-like proteins using *R. capsulatus* XDH as model. This enzyme is a cytoplasmic heterodimeric complex that catalyzes the hydroxylation of hypoxanthine and xanthine, the last two steps in purine degradation (for review, see [163]). The XdhA subunit contains two [2Fe-2S] clusters in addition to flavin adenine dinucleotide (FAD), while the XdhB subunit binds Mo-PPT (PDB ID code 1JRO) [164,165]. XdhC, encoded by the *xdhABC* operon, is essential for the production of an active and Moco-loaded *R. capsulatus* XdhAB complex [43]. Structural genomics data obtained on the XdhC homologs from *Halalkalibacterium halodurans* C-125 (formerly *Bacillus halodurans*) or *Mycobacterium smegmatis* (PDB ID codes 3ON5 and 2WE8, respectively) depict an oligomeric structure with two NAD(P)-binding Rossmann-like domains. XdhC was shown to bind Moco and Moco biosynthetic proteins, but also to protect it during the sulfuration step until its delivery to the target enzyme [140,166]. Through a collaboration between my group and the one from Silke Leimkühler, a deeper understanding of the sulfuration step was achieved [141]. This step is ensured through the interaction of Mo-MPT-loaded XdhC with NifS4, a cysteine desulfurase, and the transfer of an inorganic sulfur from L-cysteine, essentially as FdhD [141]. XdhC forms a tight complex with the Moco-free form of XdhAB until Moco insertion, and a specific interaction between XdhC and XdhB was identified [140]. Importantly, in the absence of XdhC, the heterologous expression of *Rc*XdhAB in *E. coli* resulted in the production of a stable but inactive Moco-loaded complex [140]. Under those conditions, XdhC is not required for the insertion of Mo-MPT into XDH. By analogy, YagQ, a XdhC-like chaperone, is essential for the production of an active and stable aldehyde oxidoreductase YagTSR complex in *E. coli* [167]. In contrast to *R. capsulatus* Xdh, the inactive complex produced in the absence of YagQ was shown to be devoid of Moco while loaded with FAD and two [2Fe-2S] clusters.

The assembly of the heterotetrameric enzyme *Rc*XdhAB occurs in an ordered manner through a multistep process that involves the synthesis and interaction of both subunits, the insertion of FeS clusters and FAD into XdhA, dimerization of the XdhAB complex, and insertion of sulfurated Moco into XdhB via direct contact with XdhC, resulting in an active enzyme [168]. This process is similar to those for the Nar or Dms complexes, where FeS cluster insertion precedes Moco insertion [61,95]. Hille et al. [163] hypothesized that the rotation of a small conserved domain of bovine xanthine oxidase allows access to the interior of the complex and defines a new interacting motif for the Moco insertion machinery. Clearly, it is imperative to conduct experimental investigations of the Moco insertion process, especially in cases when other metal centers may need to be accommodated beforehand.

In light of the descriptive analysis made above regarding the folding and assembly pathways of several Mo/W-enzymes, classified according to their complexity and assembly requirements, it becomes pertinent to inquire whether there are common principles that can be discerned. Additionally, it is imperative to address some remaining fundamental questions that require further investigation. Consequently, I have chosen to focus on three pivotal facets of the folding pathway.

## 6. Number of Binding Sites and Their Location Both on the Chaperone and the Target Enzyme

A consensus has been reached regarding the existence of two distinct sites on the target Mo/W-bisPGD enzymes for chaperone binding. One site is either a remnant or a Tat signal peptide when present, while the other is located in the mature region. This has been experimentally demonstrated for NarJ [60], DmsD [36,106,133], TorD [67,156] and NapD [67,129,131,133,156]. However, the situation is different when the function associated with the binding on each site has been specialized for distinct chaperones, as in the case of formate dehydrogenases. Bioinformatic analyses have revealed a systematic genetic association of *fdhE* with operons encoding periplasmic FDHs in both archaea and bacteria. Furthermore, the crucial role of FdhE for the two periplasmic FDHs in *E. coli* suggests that it likely functions to proofread, prior translocation, a correctly folded enzyme complex, which has been aided by the action of FdhD, limited to the insertion of a sulfurated cofactor [117,119]. Despite the above-mentioned consensus, there is a disparate level of information on the description of sites on both partners as well as on the stoichiometry of the chaperone–enzyme complex.

Regarding the target metalloproteins, the study of the *E. coli* nitrate reductase complex [71] provides key information about the identity of the second site for chaperone binding where cofactor insertion takes place. This study shows that a solvent-exposed salt bridge, which is conserved in all members of the Mo/W-bisPGD enzyme family, is not only important for chaperone binding (i.e., the substitution of one of the conserved residues impairs chaperone binding) but also plays a crucial role in enzyme folding after cofactor insertion. This major finding implies a conserved mechanism for cofactor insertion within the Mo/W-bisPGD family, while also highlighting the chaperone interaction site responsible for cofactor acquisition. Additionally, this suggests that the structurally unrelated FdhD, which delivers the sulfurated Moco or NapD proteins, interact in a binding region that includes this salt bridge in their respective target enzymes. In any case, one must consider different modalities in chaperone protein folding for recognition and binding to this second binding site.

Regarding the chaperones, a consensus has also been reached regarding the location of the binding site to recognize and bind the remnant or Tat signal peptide of the cognate partners. Firstly, the available crystal structures of several members of the NarJ superfamily (IPR003765), which includes TorD and DmsD as characterized members but also related ones such as TtrD, EbdD, ClrD, ArrD or YcdY [50,104], indicate a common overall all-helical fold. A wealth of experimental studies including site-directed mutagenesis, microcalorimetry, NMR, ab initio docking, molecular dynamics simulation and even site-directed spin labeling followed by electron paramagnetic resonance spectroscopy point to an elongated and hydrophobic groove but without hardly defined residues [67,93,100,130,132,152,169,170,171].

On the contrary, there is limited knowledge regarding the location of the binding site on the chaperone for the recognition of the mature domain of the partner. However, it is understood that chaperone binding allows the protein to maintain a competent folding state for the subsequent insertion of cofactors, such as an FeS cluster preceding Moco, as in most cases. Contrasting results have been reported when comparing enzymes with different folding and assembly requirements, and a report from Iobbi-Nivol’s group showed that the TorD chaperone binds Mo-bisPGD to ensure its protected transfer to the target enzyme [159]. Apart from the fact that cofactor insertion can proceed without the help of the chaperone in the Tor/Dor/Bis family, in vivo and in vitro experiments showed that *Ec*DmsD interacts with Moco biosynthetic proteins as *Ec*TorD [109]. Additionally, residues proposed to bind the GTP moieties of the cofactor are not conserved in TorD/DmsD proteins [50], while they are in the unrelated FdhD [117]. Finally, Niedzialkowska et al. reported that the heterologous production of *Sterolibacterium denitrificans* SdhD, a TorD homolog involved in the maturation of the steroid C25 hydroxylase, results in co-purification with Mo-bisPGD under anaerobic conditions in *E. coli* [172]. While the exact function of the chaperone during this step remains unclear, it is anticipated that the chaperone will interact with the same region of the metalloprotein regardless of the scenario. Several groups have identified distinct residues for either signal peptide binding or conferring activity of their cognate partner [67,159], but residues proposed to be involved in promoting activity through cofactor insertion are clustered in a distinct but not fully defined region of the chaperone protein. A definitive answer to the structural description of the recognition mode of the enzyme partner awaits further investigation, in line with the understanding of the stoichiometry of the chaperone–enzyme complex. Given that phylogenetic analysis of members of the NarJ superfamily has revealed that NarJ is likely the ancestral version that has subsequently diversified into the DmsD, TorD and YcdY types [50], one may wonder why Mo/W-bisPGD binding is not a general one. On the other hand, if Moco delivery requires the assistance of a dedicated chaperone, this raises the question of the identity of the protein responsible for this step for periplasmic nitrate reductases that also require its sulfuration, as well as how the chaperone allows the insertion of the FeS center located close to the Moco in the vast majority of cases. In this context, the study of a larger number of systems is likely to provide new answers to these unresolved questions.

The multifunctional nature of members of the NarJ superfamily raises the question of how this is achieved at a structural level. It is tempting to speculate that the ability of the chaperone to recognize and interact with distinct sites of a metalloprotein partner is based on structural flexibility. A number of studies support this hypothesis. Conformational changes upon binding the N-terminus of NarG have been reported with *Ec*NarJ using both NMR and differential scanning calorimetry [132]. Later, the combination of site-directed spin labeling followed by EPR spectroscopy and ion mobility mass spectrometry revealed distinct molecular species and conformational dynamics during the partner binding process [170]. Importantly, NarJ structural flexibility has been confirmed in vivo using in-cell EPR spectroscopy [173]. The successful development of a method for monitoring the temporal activity of NarJ in living organisms also allows for the monitoring of associated conformational changes during Nar assembly. Overall, these studies provide evidence for the existence of a conformational selection mechanism operating during the binding of the N-terminus of NarG by NarJ. At this stage, it can be inferred that the structural flexibility of the chaperone is a common feature for other members and may play a role in binding other partners at unidentified sites of the protein. However, contrasting results were obtained for *Ec*DmsD, with NMR experiments showing chemical shift perturbations resulting from complex formation with the Tat signal peptide of DmsA [174] but not confirmed by calorimetry [175]. Concerning the structurally unrelated NapD protein, a small conformational change was observed by NMR upon signal peptide binding and a hydrophobic binding interface was predicted [129,130]. The specific conformer of NarJ that is stabilized through binding with the NarG peptide and the redistribution of the protein’s conformational ensemble is an example of allostery, where the binding of a ligand at one site affects the binding of others through a change in the protein’s shape [176]. This shift in population resulting from binding the N-terminus of the Mo-enzyme may be a key factor in enabling the subsequent binding of additional partners at yet unidentified sites of the chaperone.

## 7. Specificity versus Promiscuity of the Chaperone?

The overall description of the chaperones involved in the folding and assembly of prokaryotic molybdoenzymes is not complete without addressing their specificity or promiscuity in the process. Indeed, contrasting data have been reported throughout the years, some of which suggest exquisite specificity, while others suggest promiscuity. To what extent does this questioning offer us fresh perspectives on the phenomenon and the chaperone’s functions?

Phylogenetic analyses of chaperones have consistently shown that the obtained trees do not follow the organism’s lineage [50,124]. Apart from the difficulty inherent in ascertaining the activity of a given Mo/W enzyme from sequence annotation, this observation brings into question the expected sequence co-evolution of the chaperone–partner pair. One of the reasons for these contradictory data may stem from the absence of a definition regarding the chaperone’s binding site on the partner’s core domain for cofactor insertion, as well as the non-exclusive nature of the chaperone in this process (see above). For instance, FdhD is responsible for Moco sulfuration and insertion into all three formate dehydrogenases in *E. coli*. Interestingly, *Ec*FdhD can efficiently deliver Moco to *Rc*FDH heterologously expressed in *E. coli*, while *Rc*FdhD can barely activate *Ec*FdoGHI [124]. Furthermore, using an in vitro reconstitution assay, FdhD from *E. coli* and *R. capsulatus* can replace *Ec*TorD for *Ec*TorA activity but not TorD from *S. massilia* [124,156]. Is this observation an indication of the chaperone’s lack of specificity or the limitations of such assays? Could the existence of alternative protein partners that supply inorganic sulfur to FdhD or Moco in the case of TorD and FdhD be related to specificity? In the case of FeS assembly and insertion, it is now recognized that the successful heterologous expression of a functional FeS-containing protein requires the coexpression of A-type carriers in charge of this delivery step [177]. Similarly, heterologous expression of a chaperone or a Mo-enzyme may not systematically result in a functional system owing to loss of interaction with partners. A number of studies evaluating chaperone specificity could thus be reinterpreted in light of this aspect. Additionally, the identification of crucial residues within the chaperone that determine the specificity of enzyme recognition may be hindered by their reported structural flexibility, which is often overlooked in in vitro binding assays.

At the same time, a number of pieces of evidence question the promiscuity in the folding and assembly process of prokaryotic molybdoenzymes. DmsD is essential for the maturation of three enzymes, DmsA, YnfE and YnfF both in *E. coli* and in *Salmonella* [91,92]. Similarly, both NarJ and NarW contribute to the production of active nitrate reductases A and Z in *E. coli* [31,178]. However, NarJ from *T. thermophilus* or *Bacillus subtilis* cannot substitute those from *E. coli* (Vergnes, Zakian and Magalon, unpublished results), likely due to differences in their sequences that prevent recognition of the target enzymes. Notably, Pinchbeck et al. [179] reported that NarJ is essential for NarGHI and the assimilatory nitrate reductase NasA in *Paracoccus denitrificans*, while the target enzymes display only 25% of sequence identity. Several reports indicate successful heterologous production of functional Mo/W-bisPGD enzymes when only expressing the structural genes [180,181,182], while others require the simultaneous expression of the cognate chaperone [42,183]. Lastly, halobacteria constitute a group of archaeal organisms often displaying genes encoding formate dehydrogenases while the *fdhD* gene is absent, questioning how Moco insertion and sulfuration proceed as well as the identity of the proteins involved.

The prevailing conclusion drawn from these studies is that a thorough comprehension of the metalloprotein recognition process has not yet been attained, mainly due to the limited understanding of the exact mechanism through which the chaperone enables the metalloprotein to acquire the cofactor, as wells as the diversity of chaperone structures.

## 8. How to Insert Mo/W-bisPGD?

If this question seems incongruous at first sight, after having insisted on the involvement of chaperones dedicated to the insertion of the cofactor, and perfectly illustrated the FdhD or XdhC proteins responsible for sulfurizing the cofactor before its protected transfer to the metalloprotein, it is not entirely resolved, but we do have some indications.

Firstly, we have demonstrated that the proteins involved in the final stages of biosynthesis of the Mo/W-bisPGD cofactor form a multiprotein complex [73]. Uniquely, the existence of this complex depends on the presence of synthetic intermediates. This study thus made it possible to resolve the paradox of an unstable cofactor outside the metalloprotein and the absence of proteins ensuring its storage and homeostasis, like the Moco-carrier proteins in eukaryotes (for review in this Special Issue, see [142]), by involving this complex in the transfer step to the target. This was demonstrated in 2004 by Vergnes et al. using the nitrate reductase complex in *E. coli* as a model system [72]. Proof was provided of an interaction between each of the components of this multi-protein complex and the apoenzyme. However, two conditions had to be met to observe these interactions. The first was the presence of a mature cofactor carrying a Mo atom in the cell. While the absence of interaction in the presence of a W-cofactor confirmed the absence of activity of the enzyme complex in tungstate-grown cells, it remains unknown how the metal is recognized by the target. Is the chaperone involved in this recognition? For example, the incorporation of a W-cofactor into *Ec*TorA is not very effective and leads to the accumulation of the apoprotein in the cytoplasm, in amounts comparable to those observed in the absence of TorD [48]. It remains to be seen whether the chaperone is involved in the acquisition of W-bisPGD. The second condition was the simultaneous presence of the structural partner NarH and the chaperone NarJ. It is easy to understand that an apoNarGH complex partially structured by the insertion of FeS clusters into NarH is indispensable [71]. In contrast, the indispensable presence of the chaperone for the Moco-loaded multiprotein biosynthesis complex to interact with apoNarGH has been repeatedly misinterpreted and taken as evidence of a direct interaction between NarJ and the biosynthesis proteins. The latter has not been confirmed in vitro. However, as mentioned above, the conditions required to observe these interactions (presence of a mature cofactor) cannot easily be met in vitro. Finally, the insertion of Moco requires prior insertion of the FeS cluster in NarG, both events involving the chaperone directly or indirectly [61]. At this stage, it is interesting to draw a parallel with xanthine dehydrogenase where a comparable situation is found in the sequentiality of metal center insertion [168]. This enzyme family requires a sulfurated cofactor through the action of a dedicated chaperone, XdhC, which must interact above all with the biosynthesis protein(s) [166]. Similarly, my understanding is that FdhD interacts with the multiprotein biosynthesis complex to bind Mo-bisPGD before it is sulfurated and transferred to formate dehydrogenase. In summary, the mature form of the cofactor, whatever it may be depending on the type of Mo/W enzyme, is probably supplied by a multiprotein complex that synthesizes it. The cofactor is then transferred directly to the target protein as proposed for certain enzymes in the absence of an identified chaperone or requires the intervention of the chaperone.

## 9. Concluding Remarks

Over 30 years of research on this topic has yielded extraordinary results, with the community’s intellectual stimulation and enrichment serving as guides through the comparative analysis of different systems and the varied choice of methodological approaches. These efforts have led to the development of general and common principles that govern the folding and assembly of Mo or W enzymes in prokaryotes, as illustrated in this personal perspective review. While there are still areas of uncertainty, the intervention of a chaperone appears to be specific to prokaryotic organisms and Mo/W-bisPGD enzymes, with the exception of enzymes in the xanthine dehydrogenase family that require a sulfuration step carried out by a dedicated and specialized protein. The probable existence of a number of these enzymes from the earliest forms of life on Earth to catalyze key chemical reactions, all requiring a dedicated chaperone, is striking. Definitively, the field still retains an element of mystery that stimulates further research.

## Data Availability

Not applicable.

## Abbreviations

PPT, pyranopterin; SO, sulfite oxidase; XO, xanthine oxidase; DMSO, dimethylsulfoxide; Mo/W-bisPGD, molybdenum/tungsten-bispyranopterin guanine dinucleotide; nNar, cytoplasmically oriented membrane-bound nitrate reductase; EPR, electron paramagnetic resonance; SAXS, small angle X-ray scattering; Moco, molybdenum cofactor; Tat, twin-arginine translocation; holoNarGH, active and soluble NarGH complex; apoNarGH, inactive and soluble NarGH complex with incomplete metal cofactor content; FDH, formate dehydrogenase; Nap, periplasmic nitrate reductase; ITC, isothermal titration calorimetry.
